# Institutional capacity to acquire, assess, adapt and apply evidence in Brazil: a mixed-methods study, 2024

**DOI:** 10.1590/S2237-96222026v35e20250458.en

**Published:** 2026-04-20

**Authors:** Aurelina Aguiar de Lima, Antonia Angulo-Tuesta, Eliane Maria Medeiros Leal, Jorge Otávio Maia Barreto, Everton Nunes da Silva

**Affiliations:** 1 Universidade de Brasília, Faculdade de Ciências e Tecnologias em Saúde, Brasília, DF, Brazil Universidade de Brasília Faculdade de Ciências e Tecnologias em Saúde Brasília DF Brazil; 2 Ministério da Saúde, Superintendência do Ministério da Saúde em Pernambuco, Recife, PE, Brazil Ministério da Saúde Superintendência do Ministério da Saúde em Pernambuco Recife PE Brazil; 3 Fundação Oswaldo Cruz, Escola de Governo, Brasília, DF, Brazil Fundação Oswaldo Cruz Escola de Governo Brasília DF Brazil

**Keywords:** Evidence-Informed Policy, Health Management, Qualitative Analysis, Quantitative Analysis, Brazil., Política Informada por la Evidencia, Gestión en Salud, Análisis Cualitativo, Análisis Cuantitativo, Brasil

## Abstract

**Objective:**

To evaluate the institutional capacity of the Health Ministry's Health Science, Technology and Innovation and Economic-Industrial Complex Secretariat (*Secretaria de Ciência, Tecnologia e Inovação e do Complexo Econômico-Industrial da Saúde* - SECTICS/MS) in using scientific evidence in decision-making.

**Methods:**

This was a two-phase mixed-methods study conducted between August and September 2024 in the five SECTICS/MS departments. Data were collected through questionnaires, based on the Brazilian version of the "4A Tool", which assesses four dimensions: Acquiring, Assessing, Adapting and Applying evidence, and were quantified using SPSS software (version 30).

**Results:**

Of the 15 representatives invited, 12 participated in the quantitative phase (phase 1). Of these, 4 agreed to participate in the qualitative phase (phase 2). The majority held a doctorate (67%), were civil servants (75%) and had worked for five years or more at the Ministry of Health (67%). All reported being familiar with evidence-informed policies. The "acquire" (82%) and "assess" (72%) dimensions showed high agreement, indicating consolidated institutional capacity. The "apply" dimension had (63.0%) agreement, while the "adapt" dimension had the lowest (47%), highlighting weaknesses. Participants stressed the importance of evidence for process optimization but pointed to inequalities between departments and structural limitations, such as lack of time, barriers to accessing articles and absence of knowledge translation tools.

**Conclusion:**

This study showed progress in institutional capacities to acquire and assess evidence. However, there remains a need to strengthen the adaptation and application of evidence, especially through investments in knowledge translation that bring evidence, managers and the public closer together.

## Capacidad institucional para adquirir, evaluar, adaptar y aplicar evidencia en Brasil: un estudio de métodos mixtos, 2024

Resumen

**Objetivo:**

Evaluar la capacidad institucional de la Secretaría de Ciencia, Tecnología e Innovación y del Complejo Económico-Industrial de Salud del Ministerio de Salud (SECTICS/MS) en el uso de la evidencia científica para la toma de decisiones.

**Métodos:**

Estudio bifásico de métodos mixtos, realizado entre agosto y septiembre de 2024 en los cinco departamentos de la SECTICS/MS. Los datos se recopilaron mediante cuestionarios basados ​​en la versión brasileña de la herramienta 4A, que evalúa cuatro dimensiones: Adquisición, Evaluación, Adaptación y Aplicación de la evidencia, y se cuantificaron mediante el programa SPSS (versión 30).

**Resultados:**

De los 15 representantes invitados, 12 participaron en la fase cuantitativa (fase 1). De estos, 4 aceptaron participar en la fase cualitativa (fase 2). La mayoría tenía doctorado (67,0%), ocupaba un puesto fijo (75,0%) y había trabajado durante cinco años o más en el Ministerio de Salud (67,0%). Todos reportaron estar familiarizados con políticas basadas en evidencia. Las dimensiones "adquirir" (82,0%) y "evaluar" (72,0%) mostraron un alto nivel de acuerdo, lo que indica una capacidad institucional consolidada. La dimensión "aplicar" tuvo un 63,0% de acuerdo y "adaptar" el más bajo (47,0%), destacando debilidades. Los participantes reforzaron la importancia de la evidencia para la optimización de procesos, pero señalaron desigualdades entre departamentos y limitaciones estructurales, como falta de tiempo, barreras para acceder a los artículos y ausencia de herramientas de traducción del conocimiento.

**Conclusión:**

El estudio mostró avances en las capacidades institucionales para adquirir y evaluar evidencia. Sin embargo, sigue siendo necesario fortalecer la adaptación y aplicación de la evidencia, especialmente a través de inversiones en la traducción del conocimiento que acerquen a la evidencia, a los administradores y al público.

**Ethical aspects**

This research respected ethical principles, having obtained the following approval data:

Research ethics committee Universidade de Brasília

Opinion number: 6,775,489

Approval date: 19/4/2024

Certificate of submission for ethical appraisal : 78591123.6.0000.8093

Informed consent record: The informed consent record was accepted and signed by all participants prior to data collection.

## Introduction 

Evidence-informed policies represent a structured and consolidated approach in the field of decision-making, as they seek to incorporate research results into public policy debates and internal processes within the public sector. This approach aims to improve policy formulation and program enhancement, contributing to the efficiency and quality of public interventions and, consequently, to improving the quality of life of the population ^1^. A central challenge in health policies is overcoming the know-do gap. Even with available scientific evidence, its incorporation into policy decisions is often absent or delayed, reinforcing the need for effective knowledge translation strategies ^2^.

The Brazilian Unified Health System (*Sistema Único de Saúde*, SUS) has a complex health decision-making structure, characterized by political and administrative decentralization between the three federative entities, which requires harmonization of decisions between the federal, state and municipal levels. In this context, the Health Ministry's Health Science, Technology and Innovation and Economic-Industrial Complex Secretariat (*Secretaria de Ciência, Tecnologia e Inovação e do Complexo Econômico-Industrial da Saúde*, SECTICS/MS) seeks to play a crucial role in the acquisition, assessment, adaptation and application of scientific evidence to support public policies in an equitable manner, adapted to different regional realities. This dynamic helps the country keep up with global innovations while developing solutions tailored to its own needs, strengthening the health system, improving the quality of care, as well as promoting the country's scientific and technological autonomy.

In order to promote the health of the Brazilian population, SECTICS/MS develops, implements and evaluates the National Policy on Health Science, Technology and Innovation ^3^. This Secretariat also coordinates actions to integrate scientific knowledge into the management of the SUS, focusing on improving health decisions, supporting the production of research relevant to the health system, coordinating evidence networks and promoting mechanisms that bring researchers, managers and health professionals closer together. These actions aim to strengthen the institutional capacity of the Ministry of Health in adopting evidence-informed policies.

Despite progress, studies conducted in Australia ^4^ and Brazil ^5^ show that decision-makers often prioritize personal experiences, local contexts or political interests instead of using scientific evidence ^4^^-^^6^. In Brazil, even with initiatives such as the Evidence Centers (*Núcleos de Evidências*, NEV), linked to the Evidence-informed Policy Network (EVIPNet) and created to support decision-making in the SUS, challenges persist related to the sustainability of these initiatives, articulation between research and policy and adaptation of evidence to institutional realities ^5^. This study aims to assess the institutional capacity of SECTICS/MS to use scientific evidence in decision-making.

## Methods 

Setting 

This study investigated the institutional capacity to use evidence in the SECTICS/MS decision-making process. The Secretariat is composed of five departments: Department of SUS Health Economic-Industrial Complex and Innovation; Department of Pharmaceutical Assistance and Strategic Inputs; Department of Science and Technology; Department of Health Management and Technology Incorporation; Department of Health Economics and Development ^7^.

Design

This is a mixed two-phase study, conducted between August and September 2024. Both phases were based on the Brazilian version of the 4A tool instrument, which refers to four evaluative dimensions (acquire, assess, adapt and apply) ^8^.

In phase 1 (quantitative), an online questionnaire was sent to obtain data on the characterization of the respondents' profile, such as collection of demographic data (sex and age range), human capital (education and years of experience) and knowledge about evidence-informed policies. Also at this stage, the study participants received another questionnaire containing 40 items distributed over four dimensions: acquire (12 items), assess (5 items), adapt (8 items) and apply (15 items) evidence in the decision-making process. The questions were answered individually and remotely, using a five-point Likert scale (strongly disagree = 1; disagree = 2; neither agree nor disagree = 3; agree = 4; strongly agree = 5) ^9^.

In phase 2 (qualitative), the consolidated responses from phase 1 were presented in a face-to-face meeting. Subsequently, seven guiding questions based on the 4A tool were discussed, related to the study participants' perception of barriers and facilitators for the institutional capacity to use evidence in the decision-making process. The discussions were facilitated using the focus group technique and lasted 90 minutes.

The quantitative approach was used to measure patterns related to the scientific evidence acquisition, assessment, adaptation and application dimensions, while the qualitative approach allowed for the exploration of perceptions, barriers and strategies to improve these abilities.

Participants

Participant recruitment was conducted using purposive sampling, via an official letter sent through the Electronic Information System to the SECTICS/MS Head Office. Fifteen representatives were requested to participate in the first and second phases of the study. The invitation stated that nominated participants would be required to participate in both phases: phase 1, involving the submission and completion of online questionnaires, and phase 2, conducted in person as a focus group. All participants from phase 1 were to be invited back for phase 2. Up to three representatives from each department could be nominated, following the study's eligibility criteria.

The inclusion criteria were: having an employment relationship with one of the departments of the Secretariat; having at least three months experience in the department and two years experience in the Ministry of Health; and being available to collaborate in both stages of the study. The exclusion criteria were: representatives temporarily absent during the study period; professionals whose functions are performed outside of SECTICS/MS, not experiencing the daily activities of the department; and representatives without a completed higher education degree.

Statistical analysis 

The quantitative data were analyzed using SPSS software (version 30). Participant characteristics were presented in simple frequency tables, and data derived from the 4A questions were summarized using frequency, measures of central tendency (mean and median), and dispersion (standard deviation and interquartile range). The proportion of "agree" and "strongly agree" responses was also calculated. A consensus criterion of ≥70% of responses in the categories was adopted, as recommended in the literature ^10^. Percentages equal to or greater than 70% were considered a high degree of agreement on the investigated item ^10^, i.e., the group of participants considers the item as being achieved by the institution.

Focus group analysis

The qualitative data obtained via the focus group were recorded using the Microsoft Teams platform and the automatic transcription function. Manual review was performed to ensure fidelity to the participants' statements. These were organized into emerging thematic categories through inductive analysis guided by the questions in the script based on the 4A tool ^8^. Two researchers independently coded the transcripts and compared them to the quantitative results. The content analysis technique proposed by Bardin ^11^ was used to analyze the interviews. The thematic categories were defined based on the 4A tool, considering: priority given to scientific evidence in the organization; integration of scientific evidence in the organization; use of scientific evidence; priorities for increasing research capacity; obtaining scientific evidence; assessment of scientific evidence; and linking scientific evidence to the main issues faced by those involved in the decision-making process.

Reflexivity

The authors of this study have experience in science and technology, including evidence-informed policy, health technology assessment and qualitative studies. Two authors had prior experience applying the 4A tool (acquire, assess, adapt and apply), including its validation in the Brazilian context.

## Results 

Of the 15 invited representatives, 13 nominations were received, of whom 12 agreed to participate in the first phase of the study (quantitative). Of this total, 4 participated in the second phase (qualitative). The characterization of the participants (Table 1) shows a qualified profile, as representatives who had doctorates (67%) and were civil servants (75.0%) predominated, holding coordination positions (67%) and with 10 or more years of experience at the Ministry of Health (42%); most had worked at SECTICS/MS for more than 5 years (42%).

All participants reported knowledge of evidence-informed policies. These professionals demonstrate an active role in the Secretariat's decision-making process, with half (50%) taking on multiple roles, including decision-making per se and direct influence over it.

Regarding the quantitative data, two dimensions obtained a percentage of agreement greater than 70%, suggesting consensus among participants on the existence of institutionalized capacity to "acquire" (82%) and "assess" (72%) evidence for the decision-making process. The "apply" dimension approached the study's cutoff point, with (63%) of responses representing "agree" and "strongly agree" and a median of 4 points, equivalent to the "agree" score on the Likert scale. "Adapting" scientific evidence obtained the lowest percentage (47%) and a median of 3 points, which signaled weaknesses in this dimension (Table 2).

Considering the 144 topics addressed in the "acquire" dimension, item 6 of this dimension was answered by 11 participants, totaling 143 responses (Table 2), which corresponds to (92%) consensus on the categories 'agree' or 'strongly agree' (Table 3). Also regarding this dimension, the items related to qualified staff (83%), access to scientific journals, databases and gray literature (92%), learning from colleagues (100%), and spaces for exchanging experiences with researchers (100%) stood out. The main weakness pointed out by the study participants in this dimension was the adequacy of the resources available to search for scientific evidence (Table 3).

In the "assess" dimension, consensus was observed regarding alignment between the Secretariat's priorities and scientific evidence (92%) and the team's capacity to assess critically (83%). However, limitations still persist in formalizing contracts with outside experts for assessment of scientific evidence (Table 3).

**Table 1:** Sociodemographic characteristics of the study participants. Brazil, 2024 (n=12)

Characteristic	Phase 1 (quantitative)^a^ n (%)	Phase 2 (qualitative)^b^ n (%)
Sex		
Female	6 (50)	2 (50)
Male	6 (50)	2 (50)
Age group (years)		
<41	5 (42)	3 (75)
≥41 and <50	4 (33)	1 (25)
≥51	3 (25)	0 (0)
Education level		
Postgraduate Specialization and/or Master's	4 (33)	1 (25)
Doctorate and/or Postdoc	8 (67)	3 (75)
Employment relationship		
Civil Servant	9 (75)	3 (75)
Other	3 (25)	1 (25)
Post held		
Aide	4 (33)	1 (25)
Coordinator	8 (67)	3 (75)
Average time working at the Health Ministry (years)		
2 and <5	4 (33)	1 (25)
≥5 and <10	3 (25)	1 (25)
≥10	5 (42)	2 (50)
Average time working at the Health Ministry SECTICS^c^		
3 months and <5 years	5 (42)	1 (25)
≥5 and <10 years	5 (42)	2 (50)
≥10 years	2 (17)	1 (50)
Knowledge of evidence-informed policy		
Yes	12 (100)	4 (100)
No	0 (0)	0 (0)
Decision-making level		
Influences those who decide	1 (8)	1 (25)
Takes part in decision-making	1 (8)	0 (0)
Makes decisions	2 (17)	0 (0)
Influences those who decide and takes part in decision-making	2 (17)	0 (0)
Influences those who decide, takes part in decision-making and makes decisions	6 (50)	3 (75)

a 12 people took part in Phase 1; ^b^ 4 people took part in Phase 2; ^c^SECTICS: Health Science, Technology and Innovation and Economic-Industrial Complex Secretariat.

**Table 2:** Summary of the results of applying the 4A tool in the Health Ministry's Health Science, Technology and Innovation and Economic-Industrial Complex Secretariat. Brazil, 2024 (n=12)

Dimension	Total answers	Total agree and strongly agree answers	Percentage agree and strongly agree answers	Mean (standard deviation)	Median (interquartile range)
Acquire	143	117	82	4 (0,7)	4 (3-5)
Assess	60	43	72	4 (0,8)	4 (3-5)
Adapt	96	45	47	3 (1,0)	3 (3-5)
Apply	180	114	63	4 (0,8)	4 (4-5)

**Table 3:** Map of the institutional capacities of the Health Ministry's Health Science, Technology and Innovation and Economic-Industrial Complex Secretariat to acquire and assess scientific evidence for decision-making. Brazil, 2024 (n=12)

Dimension	Topic covered	Scores 1 and 2^a^ n (%)	Score 3^b^ n (%)	Scores 4 and 5^c^ n (%)	Median (Interquartile)
Acquire	Are we capable of acquiring scientific evidence?				
	1. We have staff qualified to search for scientific evidence	0	2 (17)	10 (83)	4 (4-5)
	2. Our team has sufficient time to search for scientific evidence	0	4 (33)	8 (67)	4 (3-4)
	3. Our team is encouraged to search for scientific evidence (it is used in the decision-making process)	0	2 (17)	10 (83)	4 (4-5)
	4. Our team has resources to search for scientific evidence	1 (8)	4 (33)	7 (58)	4 (3-4)
	5. We have contracts with outside specialists who locate and monitor scientific evidence or do research for us	2 (17)	2 (17)	8 (66)	4 (3-4)
	Are we searching for scientific evidence in the right places?				
	1. We search for scientific evidence in scientific journals (subscription, internet or library access)	0	0	11 (92)	4 (4-5)
	2. We search for scientific evidence in non-periodical reports (gray literature)	0	1 (8)	11 (92)	4 (4-4)
	3. We search for scientific evidence in subscriber databases or accessing the internet	0	1 (8)	11 (92)	4 (4-5)
	4. We search for information on websites that group together and/or assess information sources	0	4 (33)	8 (67)	4 (3-5)
	5. We work with researchers in formal and informal meetings	0	0	12 (100)	4 (4-5)
	6. We get involved with researchers as hosts, partners in the decision-making process or sponsors	0	3 (25)	9 (75)	4 (3-5)
	7. We learn from colleagues through informal and formal networks to exchange ideas, experiences and best practices	0	0	12 (100)	4 (4-5)
Assess	Can we affirm that the scientific evidence is valid and of high quality?				
	1. The team has critical assessment skills and tools to assess the methodological quality of the scientific evidence	1 (8)	1 (8)	10 (83)	4 (4-5)
	2. The team has skills to assess the reliability of specific research, identifying related evidence and comparing methods and results	1 (8)	3 (25)	8 (67)	4 (3-5)
	3. Our organization has contracts with outside specialists for assessing evidence methodology, reliability and results	1 (8)	3 (25)	8 (67)	4 (3-5)
	Can we affirm that the scientific evidence is relevant and applicable?				
	1. Our team is able to demonstrate the relevance of the content of the scientific evidence in view of the organization's objectives	0	1 (8)	11 (92)	4 (4-5)
	2. Our organization has contracts with outside specialists for identifying similarities and differences between what we do and what the scientific evidence indicates	1(8)	5 (42)	6 (50)	4 (3-4)

a Scores 1 and 2 mean "I strongly disagree" and "I disagree", respectively; ^b^Score 3 means "I neither agree nor disagree"; ^c^Scores 4 and 5 mean "I agree" and "I strongly agree", respectively.

The "adapt" dimension was the least developed, ranging from 33% to 58%. The main challenges reported included difficulty in systematizing and communicating evidence in a way that is accessible to decision-makers, and limited availability of contracts with external experts (Table 4).

In the "apply" dimension, priority was given to using evidence within the organization (100%), with the team involved in discussions about evidence (75%) and resources allocated to access, adapt and apply evidence in the decision-making process (83%). However, problems were revealed in internal communication within the organization, which hinders exchange of information between the team (42%), as well as problems in management communication about the organization's strategies and priorities (67%), and problems regarding sufficient time for staff to dedicate themselves to the various stages of scientific evidence production (58%) (Table 4).

Regarding the focus group discussions, participants highlighted the importance of "giving greater priority" to scientific evidence within the organization, understanding it as an indispensable input that should be at the center of strategic decisions. Furthermore, they stressed the need to integrate evidence more frequently into work processes, in order to strengthen routines, promote collective learning and support interaction with outside researchers. Although the relevance of evidence for decision-making processes and its increasing value are recognized, its use is still inconsistent, varying according to the area and the availability of time and resources, which hinders the consolidation of an evidence-driven institutional culture.

When addressing priorities for strengthening research capacity, participants ranked the factors considered most critical, highlighting, in this order, availability of time, training and retention of qualified teams, financial resources, institutional incentives and collaboration with experts. The prioritization of these factors indicates that the challenge is not limited to structural aspects, but also involves the need to retain skilled professionals in a context of intense external competition. Regarding acquisition of scientific evidence, participants mentioned a preferred order of sources, such as: specialized journals; databases; gray literature; peer learning; researchers; and websites. However, they highlighted practical difficulties such as limited access to databases and a lack of standardization of information within the organization. Another point emphasized was the need to assess and adapt evidence more frequently, instead of only using it sporadically. Finally, there was consensus that evidence should be more frequently linked to the main issues faced by the institution, serving as a reference for the decision-making process at different levels (Table 5) and corroborating the quantitative findings of the study (Tables 3 and 4).

**Table 4:** Map of the institutional capacities of the Health Ministry's Health Science, Technology and Innovation and Economic-Industrial Complex Secretariat to adapt and apply scientific evidence for decision-making. Brazil, 2024 (n=12)

Dimension	Topic covered	Scores 1 and 2a n (%)	Score 3b n (%)	Scores 4 and 5c n (%)	Median (Interquartile)
Adapt	Are we able to summarize the results of the scientific evidence in language that is appropriate for the information user?				
	1. staff qualified to present evidence in a concise and accessible manner	3 (25)	5 (42)	4 (33)	3 [2-4]
	2. staff qualified to summarize all evidence in one document	4 (33)	4 (33)	4 (33)	3 [2-4]
	3. qualified staff that uses communication skills for the main issues of the decision-making process	2 (16)	5 (42)	5 (42)	3 [3-4]
	4. staff qualified to recommend actions to decision makers	2 (16)	5 (42)	5 (42)	3 [3-4]
	5. contracts with specialists for presenting research results in a concise and accessible manner	3 (25)	2 (17)	7 (58)	4 [2-4]
	6. contracts with specialists for summarizing research in documents	2 (17)	3 (25)	7 (58)	4 [3-5]
	7. contracts with specialists for relating results to decision maker issues	2 (17)	3 (25)	7 (58)	4 [3-5]
	8. contracts with specialists for recommending actions to decision makers	2 (17)	4 (33)	6 (50)	3 [3-5]
Apply	Do we follow the example and show how we value the use of scientific evidence?				
	1. Using scientific evidence is a priority	0	0	12 (100)	5 [4-5]
	2. Allocating resources to access, adapt and apply evidence	0	2 (17)	10 (83)	4 [4-5]
	3. Team involved in discussions about evidence and goals	1 (8)	2 (17)	9 (75)	4 [3-4]
	4. Management communicates strategies and priorities	1 (8)	3 (25)	8 (67)	4 [3-4]
	5. Internal communication ensures exchange of information	2 (16)	5 (42)	5 (42)	3 [3-4]
	6. Organizational culture values flexibility and continual improvement	1 (8)	3 (25)	8 (67)	4 [3-4]
	Within our decision-making processes is there a place for scientific evidence?				
	7. We make time available to identify research questions and consider evidence	1 (8)	4 (33)	7 (58)	4 [3-4]
	8. Team assessed option feasibility and impact	1 (8)	2 (17)	9 (75)	4 [3-4]
	9. We take into consideration recommendations of relevant evidence	1 (8)	4 (33)	7 (58)	4 [3-4]
	10. Team knows when and how decisions will be made	1 (8)	5 (42)	6 (50)	3 [3-4]
	11. Team contributes with evidence and knows how it will be used	0	6 (50)	6 (50)	3 [3-4]
	12. Team that provided evidence takes part in the discussions	1 (8)	4 (33)	7 (58)	4 [3-5]
	13. Team that provided evidence is part of the discussions	1 (8)	4 (33)	7 (58)	4 [3-5]
	14. Team receives feedback about decisions	2 (17)	4 (33)	6 (50)	3 [3-4]
	15. Team is informed about how evidence influenced decisions	2 (17)	3 (25)	7 (58)	4 [3-4]

a Scores 1 and 2 mean "I strongly disagree" and "I disagree", respectively; ^b^Score 3 means "I neither agree nor disagree"; ^c^Scores 4 and 5 mean "I agree" and "I strongly agree", respectively.

**Table 5:** Summary of qualitative evidence identified in a focus group. Brazil, 2024 (n=4)

Thematic lines (categories)	Focus group consensus	Illustrative excerpts
Priority of scientific evidence in the organization	Scientific evidence should have much more priority in the organization, being maintained as central to decision-making processes.	"There needs to be much more priority, so as not to reduce prioritization in relation to what it is at the moment." (Participant 1)
Integration of scientific evidence in the organization	Need to integrate evidence more frequently into work processes, strengthening routines and promoting collective learning.	"Scientific evidence could promote various improvements; we need to integrate it into work processes, which consequently demand research and valuing." (Participant 3)
Use of scientific evidence	Use of evidence is considered inconsistent, varying between areas and depending on availability of time and resources.	"In some processes, its use is more integrated, but in others it is not. There is no consistency: some areas use it more effectively and others do not, either due to lack of time or because it has not been incorporated into the work routine." (Participant 2)
Priorities for increasing research capacity	Priority hierarchy identified: 1 - better response time; 2 - qualified team; 3 - financial resources; 4º incentive; 5º outside specialists.	"We don't have enough time to produce at the pace the manager needs." (Participant 4) "Our problem isn't just making contracts with outside specialists, but maintaining a qualified team, because we train the people and the industry hires them." (Participant 4) "If we had the financial resources, we could hire external institutions." (Participant 1) "What we need least right now is incentive, because I see an internal willingness to increase research capacity." (Participant 3)
Obtaining scientific evidence	Order of most used sources: 1 - specialized journals; 2 - databases; 3 - gray literature; 4 - learning from colleagues; 5 - researchers; 6º websites. Difficulties in access and lack of standardization were reported.	"There is no standardization of information within the organization; not everyone has access to the same databases and information systems." (Participant 4) "DataSUS doesn't allows us to download articles, I download them at home." (Participant 3)
Assessment of scientific evidence	Need to assess and adapt evidence more frequently, and not just use them on a one-off basis.	"We need to assess and adapt scientific evidence more frequently." (Participant 3)
Relating evidence to the main issues faced	Evidence should be taken into consideration more frequently in decision-making processes at all levels.	"It is essential to consider scientific evidence more frequently in the decision-making process." (Participant 2)

## Discussion 

This study revealed that SECTICS/MS has consolidated institutional capacity in the dimensions of acquiring and assessing scientific evidence to support decision-making. However, significant weaknesses were identified in the "adapt" and "apply" dimensions, especially in the components of knowledge translation and communication between technical areas of the institution. The qualitative analysis pointed to heterogeneity between departments in the use of evidence, with important variations in the availability of qualified staff, time and limited access to specialized databases. Furthermore, low cooperation with outside experts was observed, hindering the practical application of scientific knowledge.

With regard to the focus group, participants pointed out relevant issues existing in Brazil such as lack of time for managers, scarcity of resources for research funding and absence of the use of research results to guide policies, which corroborates other findings ^12^^,^^13^. In fact, public investments in research and development have been reduced in Brazil, and this hinders the development of new research and the continuity of much research in progress ^12^^-^^15^. While South Korea and Israel, in 2023, invested more than 4% of their gross domestic product in research, Europe applied about 2%, the United States 3%, while Brazil allocated only 1% ^16^. It should be noted, however, that globally, approximately 80% of countries allocate less than 1% of their GDP to research and development (R&D), according to the UNESCO Science Report 2021. This highlights the significant inequality in the global distribution of scientific investment efforts ^17^. These elements contribute to a perception of inconsistent and poorly systematized use of scientific evidence in the formulation of public policies.

The main limitation of this study was the low participation in phase 2, with only three of the five SECTICS/MS departments participating, which reduced the scope of the perspectives. Furthermore, restricting the study exclusively to SECTICS/MS limited the generalizability of the results to other bodies within the Ministry of Health and other institutional contexts.

The selection of participants was done by convenience, through nomination, which may have generated selection bias, restricting the diversity of profiles and opinions. The invitation method may have favored the participation of individuals closer to or already engaged with the topic. Alternatives such as conducting anonymous surveys and including other sectors and institutions could have broadened the participation and representativeness of the sample, which would possibly influence the results obtained. There was also no longitudinal analysis, which limits evaluation of changes over time. The self-administered nature of the instrument may have generated response bias, such as social desirability. To mitigate these effects, measures such as anonymous answers, clarity in the formulation of items and standardization of instructions for participants were adopted. Finally, one participant did not answer an item in the "acquire" dimension; however, this missing response did not alter the result of that item, as the other 11 participants responded "agree" or "strongly agree", reaching 92% consensus.

The findings are consistent with national and international studies that apply the 4A tool, which also indicate greater capacity to acquire evidence and difficulties in the "adapt" and "apply" stages ^4^^,^^5^^,^^18^. In 2013, institutions in Canada showed high capacity to acquire evidence and moderate capacity to assess and adapt, but faced challenges in applying the evidence in practice ^18^. In Burkina Faso, Canada and Nigeria, in 2007, 2011 and 2015 respectively, these capacities were limited in all dimensions, with more significant challenges in assessment, adaptation and application of evidence ^19^^-^^21^.

In 2020, in Brazil, the Evidence Centers were observed to have adequate technical qualifications to acquire and assess evidence, but also faced challenges to their sustainability, especially in strengthening "adaptation" and "application" ^5^.

Knowledge translation is a persistent barrier, especially in low- and middle-income countries, which face shortages of human and financial resources ^19^. Difficulty in accessing evidence (research and closed databases), scarcity of qualified staff, little interaction with researchers and insufficient resources are the main bottlenecks faced in this area ^22^. However, it is easier to implement research when training on the use of evidence is received ^23^^,^^24^.

Several global initiatives with implications for Brazil have been implemented, namely: 

The EVIPNet network was created in 2005, promoted by the World Health Organization, with the aim of facilitating the sharing of best practices and connecting researchers and decision-makers, with repercussions in Brazil in 2007, with the creation of the network for evidence-informed policies ^25^; 

Evidence syntheses have been developed, which produce summaries from systematic reviews that translate scientific knowledge into a format accessible and applicable to local contexts, such as the first synthesis published in Brazil in 2010 ^26^; 

Institutional capacities have been strengthened through training programs for health professionals and managers in evidence analysis and practical application, such as the postgraduate specialization course in evidence-informed policies in Brazil ^27^^,^^28^; 

Open knowledge platforms have been established, providing open repositories and databases to facilitate access to scientific information, such as the Cochrane Library, Health Evidence and Health systems Evidence; 

Government policies have been adopted, focusing on establishing norms and regulations that require use of scientific evidence in the formulation and evaluation of public policies, such as Law No. 12401/2011 on the incorporation of technologies into the Brazilian Unified Health System; 

Research initiatives that directly connect scientific research with public health issues have been encouraged. These initiatives can be expanded to benefit the entire country ^23^^,^^24^.

The perception revealed by the quantitative and qualitative findings, that scientific evidence, while valuable, is not yet used consistently or frequently enough in decision-making processes, reflects the need for greater investment in the institutionalization of evidence-informed policies and knowledge translation, in order to promote strategies to overcome barriers that hinder integration between scientific evidence, policymakers and the public.

Creating a methodological support center strengthens the systematization and communication of scientific evidence. Expanded access to scientific databases and evidence synthesis tools enhances the use of knowledge. Intersectoral integration promotes the sharing of best practices. Formal guidelines for knowledge translation support the adaptation of evidence to the decision-making context. These strategies can contribute to the institutionalization of evidence-informed policies. Investing in the valuing of science and strengthening institutional arrangements that promote the use of evidence in public policies is fundamental to strengthening the Brazilian Unified Health System. Continued studies in this area will contribute to consolidating evidence-informed practices, expanding the positive impact on the formulation and implementation of public policies in Brazil.

## Data Availability

The database and the analysis codes used in this research are available from the authors upon request.
