# Supplementary material for: Institutional capacity to acquire, assess, adapt and apply evidence in Brazil: a mixed-methods study, 2024
Source: Epidemiol Serv Saude. 2026 Apr 20;35:e20250458. doi: 10.1590/S2237-96222026v35e20250458.en (PMC13686393; doi:10.1590/S2237-96222026v35e20250458.en)
Supplement: Translation

# Translation: Capacidade institucional para adquirir, avaliar, adaptar e aplicar evidências no Brasil: estudo misto, 2024

**DOI:** 10.1590/S2237-96222026v35e20250458.pt

**Authors:** Aurelina Aguiar de Lima, Antonia Angulo-Tuesta, Eliane Maria Medeiros Leal, Jorge Otávio Maia Barreto, Everton Nunes da Silva

**Affiliations:** 1 Universidade de Brasília, Faculdade de Ciências e Tecnologias em Saúde, Brasília, DF, Brasil Universidade de Brasília Faculdade de Ciências e Tecnologias em Saúde Brasília DF Brasil; 2 Ministério da Saúde, Superintendência do Ministério da Saúde em Pernambuco, Recife, PE, Brasil Ministério da Saúde Superintendência do Ministério da Saúde em Pernambuco Recife PE Brasil; 3 Fundação Oswaldo Cruz, Escola de Governo, Brasília, DF, Brasil Fundação Oswaldo Cruz Escola de Governo Brasília DF Brasil

**Keywords:** Política Informada por Evidências, Gestão em Saúde, Análise Qualitativa, Análise Quantitativa, Brasil.

## Abstract

**Objetivo:**

Avaliar a capacidade institucional da Secretaria de Ciência, Tecnologia e Inovação e do Complexo Econômico-Industrial da Saúde do Ministério da Saúde (SECTICS/MS) em utilizar evidências científicas na tomada de decisão.

**Métodos:**

Estudo misto de duas fases realizado entre agosto e setembro de 2024 nos cinco departamentos da SECTICS/MS. Os dados foram coletados através de questionários, baseados na versão brasileira da "Ferramenta 4A", que avalia quatro dimensões: Adquirir, Avaliar, Adaptar e Aplicar evidências e foram quantificados utilizando o software SPSS (versão 30).

**Resultados:**

Dos 15 representantes convidados, 12 participaram da fase quantitativa (fase 1). Destes, 4 aceitaram participar da fase qualitativa (fase 2). A maioria possuía doutorado (67%), vínculo estatutário (75%) e atuava há cinco anos ou mais no Ministério da Saúde (67%). Todos relataram conhecer as políticas informadas por evidências. As dimensões "adquirir" (82%) e "avaliar" (72%) obtiveram alta concordância, o que indicou capacidade institucional consolidada. A dimensão "aplicar" teve (63,0%) e a "adaptar", o menor índice (47%), evidenciando fragilidades. Os participantes reforçaram a importância das evidências para a otimização de processos, mas apontaram desigualdade entre departamentos e limitações estruturais, como falta de tempo, barreiras de acesso a artigos e ausência de ferramentas de tradução do conhecimento.

**Conclusão:**

O estudo evidenciou avanços nas capacidades institucionais de adquirir e avaliar evidências. No entanto, persiste a necessidade de fortalecer a adaptação e aplicação das evidências, especialmente por meio de investimentos em tradução do conhecimento que aproximem evidências, gestores e população.

**Aspectos éticos**

Esta pesquisa respeitou os princípios éticos, obtendo os seguintes dados de aprovação:

Comitê de ética em pesquisa: Universidade de Brasília

Número do parecer: 6.775.489

Data de aprovação: 19/4/2024

Certificado de apresentação de apreciação ética: 78591123.6.0000.8093

Registro do consentimento livre e esclarecido: O termo livre e esclarecido foi aceito e assinado por todos os participantes antes da coleta.

## Introdução 

As políticas informadas por evidências representam uma abordagem estruturada e consolidada no campo da tomada de decisão, pois buscam incorporar resultados de pesquisas nos debates de políticas públicas e nos processos internos do setor público. Essa abordagem visa aprimorar a formulação de políticas e a melhoria de programas, o que contribui para a eficiência e a qualidade das intervenções públicas e, consequentemente, para a melhoria da qualidade de vida da população ^1^. Um desafio central nas políticas de saúde é superar o abismo entre o saber e o fazer (*know-do gap*). Mesmo com evidências científicas disponíveis, sua incorporação nas decisões políticas é frequentemente ausente ou retardada, o que reforça a necessidade de estratégias efetivas de tradução do conhecimento ^2^. 

O Sistema Único de Saúde (SUS) no Brasil conta com organização complexa de tomada de decisão em saúde, caracterizado pela descentralização política-administrativa entre os três entes federativos que exige a harmonização das decisões entre os níveis federal, estadual e municipal. Nesse contexto, a Secretaria de Ciência, Tecnologia e Inovação e do Complexo Econômico-Industrial da Saúde do Ministério da Saúde (SECTICS/MS) busca desempenhar papel crucial na aquisição, avaliação, adaptação e aplicação de evidências científicas para subsidiar políticas públicas de forma equitativa e adaptada às diferentes realidades regionais. Essa dinâmica contribui para que o país acompanhe inovações globais, ao mesmo tempo em que desenvolve soluções adaptadas às suas próprias necessidades, fortalecendo o sistema de saúde, melhorando a qualidade da assistência e promovendo a autonomia científica e tecnológica do país.

Para promover a saúde da população brasileira, a SECTICS/MS elabora, implementa e avalia a Política Nacional de Ciência, Tecnologia e Inovação em Saúde ^3^. Essa secretaria também coordena ações para integrar o conhecimento científico à gestão do SUS, com foco na qualificação das decisões em saúde, no apoio à produção de pesquisas relevantes para o sistema de saúde, na coordenação de redes de evidências e na promoção de mecanismos que aproximem pesquisadores, gestores e profissionais de saúde. Essas ações visam fortalecer a capacidade institucional do Ministério da Saúde na adoção de políticas informadas em evidências.

Apesar dos avanços, estudos realizados na Austrália ^4^ e no Brasil ^5^ mostram que os tomadores de decisão frequentemente priorizam experiências pessoais, contextos locais ou interesses políticos em vez de utilizar evidências científicas ^4^^-^^6^. No Brasil, mesmo com iniciativas como os Núcleos de Evidências (Nev), vinculados à Rede para Políticas Informadas por Evidências (*Evidence-informed Policy* Network, EVIPNet) e criados para apoiar decisões no SUS, persistem desafios relacionados à sustentabilidade dessas iniciativas, à articulação entre pesquisa e política, e à adaptação das evidências às realidades institucionais ^5^. Este estudo tem como objetivo avaliar a capacidade institucional da SECTICS/MS em utilizar evidências científicas na tomada de decisão. 

## Métodos 

Contexto do estudo 

Este estudo investigou a capacidade institucional em usar evidências no processo decisório da SECTICS/MS que é composta de cinco departamentos, sendo eles: Departamento do Complexo Econômico-Industrial da Saúde e de Inovação para o SUS, Departamento de Assistência Farmacêutica e Insumos Estratégicos, Departamento de Ciência e Tecnologia, Departamento de Gestão e Incorporação de Tecnologias em Saúde e Departamento de Economia e Desenvolvimento em Saúde ^7^.

Desenho do estudo

Estudo misto de duas fases, realizado entre agosto e setembro de 2024. As duas fases foram baseadas no instrumento ferramenta 4A, versão brasileira, fazendo referência às quatro dimensões avaliativas (adquirir, avaliar, adaptar e aplicar) ^8^.

Na fase 1 (quantitativa), foi enviado um questionário on-line para obter os dados sobre a caracterização do perfil dos respondentes, como a coleta de dados demográficos (sexo e faixa etária), capital humano (formação e tempo de experiência) e conhecimento sobre políticas informadas por evidências. Ainda nesse momento, os participantes do estudo receberam outro questionário contendo 40 itens distribuídos em quatro dimensões: adquirir (12 itens), avaliar (5 itens), adaptar (8 itens) e aplicar (15 itens) evidências no processo de tomada de decisão. As questões foram respondidas de forma individual e remota, por meio de uma escala Likert de cinco pontos (discordo fortemente = 1; discordo = 2; não concordo nem discordo = 3; concordo = 4; concordo plenamente = 5) ^9^.

Na fase 2 (qualitativa), foi apresentada a consolidação das respostas da fase 1 em reunião presencial. Posteriormente, foram discutidas sete questões norteadoras baseadas no instrumento ferramenta 4A relacionadas à percepção dos participantes do estudo sobre barreiras e facilitadores da capacidade institucional em usar evidências no processo de tomada de decisão. As discussões foram fomentadas com base na técnica de grupo focal com duração de 90 minutos.

A abordagem quantitativa foi empregada para mensurar padrões relacionados às dimensões de aquisição, avaliação, adaptação e aplicação de evidências científicas, enquanto a qualitativa permitiu explorar percepções, barreiras e estratégias para aprimorar essas capacidades.

Participantes

O recrutamento dos participantes foi realizado com amostragem intencional, por meio de ofício enviado via Sistema Eletrônico de Informações ao Gabinete da SECTICS/MS. Solicitou-se a indicação de 15 representantes para participarem da primeira e da segunda fases do estudo. No convite, foi informado que os participantes indicados deveriam integrar as duas fases do estudo: a fase 1, referente ao envio e ao preenchimento de questionários on-line, e a fase 2, realizada presencialmente na forma de grupo focal. Todos os participantes da fase 1 seriam novamente convidados para a fase 2. Poderia haver a indicação de até três representantes de cada departamento, seguindo os critérios de elegibilidade do estudo. 

Os critérios de inclusão foram: ter vínculo de trabalho com um dos departamentos da secretaria; ter experiência de pelo menos três meses no departamento e de dois anos no Ministério da Saúde e ter disponibilidade para colaborar nas duas etapas do estudo. Os critérios de exclusão foram: representantes temporariamente afastados no período de execução do estudo; profissionais cujas funções são desenvolvidas externas à SECTICS/MS, não vivenciando as atividades cotidianas do departamento e representantes sem ensino superior completo. 

Análise estatística 

Os dados quantitativos foram analisados utilizando o software SPSS (versão 30). As características dos participantes foram apresentadas em tabelas de frequência simples, e os dados das questões 4A foram sumarizados por meio de frequência, medidas de tendência central (média e mediana) e dispersão (desvio- padrão e intervalo interquartil). Também foi calculada a proporção de respostas "concordo" e "concordo plenamente". Adotou-se como critério de consenso o percentual ≥70% de respostas nas categorias, conforme recomendado na literatura ^10^. Percentuais iguais ou superiores a 70% foram considerados como alto grau de concordância sobre o item investigado ^10^, ou seja, o grupo de participantes considera o item atingido pela instituição.

Análise do grupo focal

Os dados qualitativos provenientes do grupo focal foram gravados pela plataforma Microsoft Teams*,* usando a função de transcrição automática. Foi realizada revisão manual para garantir fidelidade às falas dos participantes. Estas foram organizadas em categorias temáticas emergentes, por meio de análise indutiva guiada pelas perguntas do roteiro baseado na ferramenta 4A ^8^. Dois pesquisadores codificaram as transcrições de forma independente e compararam aos resultados quantitativos. Para análise das entrevistas, utilizou-se a técnica de análise de conteúdo, proposta por Bardin ^11^. As categorias temáticas foram definidas com base na ferramenta dos 4 As, considerando: prioridade das evidências científicas na organização; integração das evidências científicas na organização; uso de evidências científicas; prioridades para aumentar a capacidade de pesquisa; obtenção de evidências científicas; avaliação das evidências científicas e vinculação das evidências científicas às principais questões enfrentadas por aqueles envolvidos no processo de tomada de decisão. 

Reflexividade 

Os autores que subscreveram este estudo têm experiência em ciência e tecnologia, incluindo políticas informadas por evidências, avaliação de tecnologias em saúde e estudos qualitativos. Dois autores tiveram experiência prévia com a aplicação da ferramenta 4A (adquirir, avaliar, adaptar e aplicar), inclusive com sua validação ao contexto brasileiro. 

## Resultados 

Dos 15 representantes convidados, foram recebidas 13 indicações, das quais 12 aceitaram participar da primeira fase do estudo (quantitativa). Deste montante, 4 participaram na segunda fase (qualitativa). A caracterização dos participantes (Tabela 1), evidencia um perfil qualificado, pois predominaram representantes com doutorado (67%) e vínculo estatutário (75%), em cargo de coordenação (67%) e com atuação igual ou superior a 10 anos no Ministério da Saúde (42%); a maior parte atuava na SECTICS/MS por mais de 5 anos (42%). 

Todos os participantes relataram conhecimento sobre políticas informadas por evidências. Esses profissionais demonstram papel ativo no processo de decisão da secretaria, com metade (50%) assumindo múltiplas funções, incluindo a tomada de decisão *per se* e a influência direta sobre elas. 

Em relação aos dados quantitativos, duas dimensões obtiveram percentual de concordância superior a 70%, o que sugere consenso dos participantes sobre a existência de capacidade institucionalizada para "adquirir" (82%) e "avaliar" (72%) evidências para o processo de tomada de decisão. A dimensão "aplicar" aproximou-se do ponto de corte do estudo, com (63%) das respostas representando "concordo" e "concordo plenamente" e mediana de 4 pontos, equivalente à pontuação de "concordo" na escala Likert. "Adaptar" evidências científicas obteve o menor percentual (47%) e mediana de 3 pontos, o que sinalizou fragilidades nessa dimensão (Tabela 2). 

Ao considerar os 144 tópicos abordados na dimensão "adquirir", o item 6 dessa dimensão foi respondido por 11 participantes, totalizando 143 respostas (Tabela 2), o que corresponde a (92%) de consenso sobre as categorias 'concordo' ou 'concordo plenamente (Tabela 3). Ainda sobre essa dimensão, destacaram-se os itens relacionados à equipe qualificada (83%), ao acesso a revistas científicas, aos bancos de dados e literatura cinzenta (92%), à aprendizagem com colegas (100%) e aos espaços de troca de experiência com pesquisadores (100%). A principal fragilidade apontada pelos participantes do estudo nessa dimensão foi a adequação dos recursos disponíveis para buscar evidências científicas (Tabela 3). 

**Tabela 1:** Características sociodemográficas dos participantes do estudo. Brasil, 2024 (n=12)

Característica	Fase 1 (quantitativo)a n (%)	Fase 2 (qualitativo)b n (%)
Sexo		
Feminino	6 (50)	2 (50)
Masculino	6 (50)	2 (50)
Faixa etária (anos)		
<41	5 (42)	3 (75)
≥41 e <50	4 (33)	1 (25)
≥51	3 (25)	0 (0)
Nível de escolaridade		
Especialização e/ou mestrado	4 (33)	1 (25)
Doutorado e/ou pós-doutorado	8 (67)	3 (75)
Vínculo empregatício		
Servidor público	9 (75)	3 (75)
Outros	3 (25)	1 (25)
Cargo que ocupa		
Assessoria	4 (33)	1 (25)
Coordenação	8 (67)	3 (75)
Tempo médio de atuação no Ministério da Saúde (anos)		
2 e <5	4 (33)	1 (25)
≥5 e <10	3 (25)	1 (25)
≥10	5 (42)	2 (50)
Tempo médio de atuação na SECTICSc do Ministério da Saúde		
3 meses e <5 anos	5 (42)	1 (25)
≥5 e <10 anos	5 (42)	2 (50)
≥10 anos	2 (17)	1 (50)
Conhecimento em política informada por evidências		
Sim	12 (100)	4 (100)
Não	0 (0)	0 (0)
Nível na tomada de decisão		
Influência quem decide	1 (8)	1 (25)
Participa da tomada de decisão	1 (8)	0 (0)
Toma decisões	2 (17)	0 (0)
Influência quem decide e participa da tomada de decisão	2 (17)	0 (0)
Influência quem decide, participa da tomada de decisão e toma decisão	6 (50)	3 (75)

a 12 pessoas participaram da fase 1; ^b^ 4 pessoas participaram da fase 2; ^c^SECTICS: Secretaria de Ciência, Tecnologia e Inovação e do Complexo Econômico-Industrial da Saúde.

**Tabela 2:** Síntese dos resultados da aplicação da ferramenta 4A na Secretaria de Ciência, Tecnologia e Inovação e do Complexo Econômico-Industrial da Saúde do Ministério da Saúde. Brasil, 2024 (n=12)

Dimensões	Total de respostas	Total de respostas concordo e concordo plenamente	Percentual de respostas concordo e concordo plenamente	Média (desvio-padrão)	Mediana (intervalo interquartil)
Adquirir	143	117	82	4 (0,7)	4 (3-5)
Avaliar	60	43	72	4 (0,8)	4 (3-5)
Adaptar	96	45	47	3 (1,0)	3 (3-5)
Aplicar	180	114	63	4 (0,8)	4 (4-5)

**Tabela 3:** Mapeamento das capacidades institucionais da Secretaria de Ciência, Tecnologia e Inovação e do Complexo Econômico-Industrial da Saúde do Ministério da Saúde em adquirir e avaliar evidências científicas para tomada de decisão. Brasil, 2024 (n=12)

Dimensão	Tópico abordado	Escores 1 e 2^a^ n (%)	Escore 3^b^ n (%)	Escores 4 e 5^c^ n (%)	Mediana (Interquartil)
Adquirir	Somos capazes de adquirir evidências científicas?				
	1. Temos pessoal qualificado para realizar busca de evidências científicas	0	2 (17)	10 (83)	4 [4-5]
	2. Nossa equipe tem tempo suficiente para realizar busca de evidências científicas	0	4 (33)	8 (67)	4 [3-4]
	3. Nossa equipe tem incentivo para buscar evidências científicas (elas são usadas no processo de tomada de decisão)	0	2 (17)	10 (83)	4 [4-5]
	4. Nossa equipe tem recursos para buscar evidências científicas	1 (8)	4 (33)	7 (58)	4 [3-4]
	5. Temos acordos com especialistas externos que localizam e monitoram evidências científicas ou conduzem pesquisas para nós	2 (17)	2 (17)	8 (66)	4 [3-4]
	Estamos buscando as evidências científicas nos lugares corretos?				
	1. Buscamos evidências científicas em revistas científicas (assinatura, acesso à internet ou biblioteca)	0	0	11 (92)	4 [4-5]
	2. Buscamos evidências científicas em relatórios não periódicos (literatura cinzenta)	0	1 (8)	11 (92)	4 [4-4]
	3. Buscamos evidências científicas em bancos de dados por assinatura ou acesso à internet	0	1 (8)	11 (92)	4 [4-5]
	4. Buscamos informações em sites que agrupam e/ou avaliam fontes de informação	0	4 (33)	8 (67)	4 [3-5]
	5. Trabalhamos com pesquisadores em reuniões formais e informais	0	0	12 (100)	4 [4-5]
	6. Nos envolvemos com pesquisadores como anfitriões, parceiros no processo de tomada de decisão ou patrocinadores	0	3 (25)	9 (75)	4 [3-5]
	7. Aprendemos com colegas através de redes informais e formais para trocar ideias, experiências e melhores práticas	0	0	12 (100)	4 [4-5]
Avaliar	Podemos afirmar que as evidências científicas são válidas e de alta qualidade?				
	1. A equipe possui habilidades de avaliação crítica e ferramentas para avaliar a qualidade metodológica das evidências científicas	1 (8)	1 (8)	10 (83)	4 [4-5]
	2. A equipe possui habilidades para avaliar a confiabilidade de pesquisas específicas, identificando evidências relacionadas e comparando métodos e resultados	1 (8)	3 (25)	8 (67)	4 [3-5]
	3. Nossa organização possui acordos com especialistas externos para avaliar metodologia, confiabilidade e resultados das evidências	1 (8)	3 (25)	8 (67)	4 [3-5]
	Podemos afirmar que as evidências científicas são relevantes e aplicáveis?				
	1. Nossa equipe pode demonstrar a relevância do conteúdo das evidências científicas frente aos objetivos da organização	0	1 (8)	11 (92)	4 [4-5]
	2. Nossa organização possui acordos com especialistas externos para identificar semelhanças e diferenças entre o que praticamos e o que indicam as evidências científicas	1(8)	5 (42)	6 (50)	4 [3-4]

^a^ Os escores 1 e 2 significam "discordo plenamente" e "discordo", respectivamente; ^b^O escore 3 significa "nem concordo e nem discordo"; ^c^Os escores 4 e 5 significam "concordo" e "concordo plenamente", respectivamente.

Na dimensão "avaliar", observaram-se consenso sobre o alinhamento entre as prioridades da secretaria e as evidências científicas (92%) e capacidade de avaliação crítica da equipe (83%). No entanto, ainda persistem limitações na formalização de acordos com especialistas externos para avaliação de evidências científicas (Tabela 3). 

A dimensão "adaptar" foi a menos desenvolvida e variou entre 33% e 58%. Os principais desafios relatados incluíram a dificuldade em sistematizar e comunicar evidências de forma acessível aos tomadores de decisão e a limitação de acordos com especialistas externos (Tabela 4). 

Na dimensão "aplicar", reconheceu-se a prioridade em usar evidências na organização (100%), com equipe envolvida nas discussões sobre evidências (75%) e alocação de recursos para acessar, adaptar e aplicar evidências no processo de tomada de decisão (83%). Porém, revelaram-se problemas de comunicação interna à organização, o que dificulta a troca de informação entre a equipe (42%), de comunicação da gerência sobre as estratégias e prioridades da organização (67%), e de tempo suficiente para dedicar-se às várias etapas da produção de evidências científicas (58%) (Tabela 4).

Com relação às discussões em grupo focal, os participantes destacaram a importância de "atribuir maior prioridade" às evidências científicas dentro da organização, entendendo-as como insumos indispensáveis que devem estar no centro das decisões estratégicas. Além disso, reforçaram a necessidade de integrar as evidências com maior frequência aos processos de trabalho, de modo a fortalecer rotinas, promover aprendizado coletivo e apoiar a interação com pesquisadores externos. Embora se reconheça a relevância das evidências para os processos decisórios e sua crescente valorização, observou-se que seu uso ainda ocorre de maneira inconsistente, variando conforme a área e a disponibilidade de tempo e recursos, o que dificulta a consolidação de uma cultura institucional orientada por evidências.

Ao abordar as prioridades para o fortalecimento da capacidade de pesquisa, os participantes hierarquizaram os fatores considerados mais críticos, destacando, nessa ordem, a disponibilidade de tempo, a formação e retenção de equipes qualificadas, os recursos financeiros, os incentivos institucionais e a colaboração com especialistas. A priorização desses fatores indica que o desafio não se limita a aspectos estruturais, mas envolve também a necessidade de manter profissionais capacitados em um contexto de intensa competição externa. Quanto à obtenção de evidências científicas, os participantes mencionaram uma ordem preferencial de fontes, tais como: revistas especializadas; bancos de dados; literatura cinzenta; aprendizado entre colegas; pesquisadores e websites. Contudo, eles ressaltaram dificuldades práticas como acesso limitado a bases e falta de padronização da informação dentro da organização. Outro ponto enfatizado foi a necessidade de avaliar e adaptar as evidências com maior frequência, em vez de apenas utilizá-las de forma pontual. Por fim, houve consenso de que as evidências devem ser mais frequentemente vinculadas às principais questões enfrentadas pela instituição, servindo de referência para o processo decisório em diferentes níveis (Tabela 5) corroborando com os achados quantitativos do estudo (Tabelas 3 e 4).

**Tabela 4:** Mapeamento das capacidades institucionais da Secretaria de Ciência, Tecnologia e Inovação e do Complexo Econômico-Industrial da Saúde do Ministério da Saúde em adaptar e aplicar evidências científicas para tomada de decisão. Brasil, 2024 (n=12)

Dimensão	Tópico abordado	Escores 1 e 2^a^ n (%)	Escore 3^b^ n (%)	Escores 4 e 5^c^ n (%)	Mediana (Interquartil)
Adaptar	Podemos resumir os resultados das evidências científicas em linguagem adequada ao usuário da informação?				
	1. pessoal qualificado para apresentar evidências de forma concisa e acessível	3 (25)	5 (42)	4 (33)	3 (2-4)
	2. pessoal qualificado para sintetizar em documento todas as evidências	4 (33)	4 (33)	4 (33)	3 (2-4)
	3. pessoal qualificado que usa habilidades de comunicação para principais questões do processo decisório	2 (16)	5 (42)	5 (42)	3 (3-4)
	4. pessoal qualificado para recomendar ações aos tomadores de decisão	2 (16)	5 (42)	5 (42)	3 (3-4)
	5. acordos com especialistas para apresentar resultados de pesquisa concisos e acessíveis	3 (25)	2 (17)	7 (58)	4 (2-4)
	6. acordos com especialistas para sintetizar pesquisas em documentos	2 (17)	3 (25)	7 (58)	4 (3-5)
	7. acordos com especialistas para vincular resultados às questões dos tomadores de decisão	2 (17)	3 (25)	7 (58)	4 (3-5)
	8. acordos com especialistas para recomendar ações aos tomadores de decisão	2 (17)	4 (33)	6 (50)	3 (3-5)
Aplicar	Seguimos o exemplo e mostramos como valorizamos o uso das evidências científicas?				
	1. Usar evidências científicas é prioridade	0	0	12 (100)	5 (4-5)
	2. Alocação de recursos para acessar, adaptar e aplicar evidências	0	2 (17)	10 (83)	4 (4-5)
	3. Equipe envolvida em discussões sobre evidências e metas	1 (8)	2 (17)	9 (75)	4 (3-4)
	4. Gerência comunica estratégias e prioridades	1 (8)	3 (25)	8 (67)	4 (3-4)
	5. Comunicação interna garante troca de informações	2 (16)	5 (42)	5 (42)	3 (3-4)
	6. Cultura organizacional valoriza flexibilidade e melhoria contínua	1 (8)	3 (25)	8 (67)	4 (3-4)
	Dentro dos nossos processos de tomada de decisão existe um lugar para as evidências científicas?				
	7. Disponibilizamos tempo para identificar perguntas de pesquisa e considerar evidências	1 (8)	4 (33)	7 (58)	4 (3-4)
	8. Equipe avalia viabilidade e impacto de opções	1 (8)	2 (17)	9 (75)	4 (3-4)
	9. Consideramos recomendações de evidências relevantes	1 (8)	4 (33)	7 (58)	4 (3-4)
	10. Equipe sabe quando e como decisões serão tomadas	1 (8)	5 (42)	6 (50)	3 (3-4)
	11. Equipe contribui com evidências e sabe como serão usadas	0	6 (50)	6 (50)	3 (3-4)
	12. Equipe que forneceu evidências participa das discussões	1 (8)	4 (33)	7 (58)	4 (3-5)
	13. Equipe que forneceu evidências faz parte das discussões	1 (8)	4 (33)	7 (58)	4 (3-5)
	14. Equipe recebe feedback sobre decisões	2 (17)	4 (33)	6 (50)	3 (3-4)
	15. Equipe é informada de como evidências influenciaram decisões	2 (17)	3 (25)	7 (58)	4 (3-4)

^a^ Os escores 1 e 2 significam "discordo plenamente" e "discordo", respectivamente; ^b^O escore 3 significa "nem concordo nem discordo"; ^c^Os escores 4 e 5 significam "concordo" e "concordo plenamente", respectivamente.

**Tabela 5:** Síntese das evidências qualitativas identificadas em grupo focal. Brasil, 2024 (n=4)

Linhas temáticas (categorias)	Consenso do grupo focal	Trechos ilustrativos
Prioridade das evidências científicas na organização	As evidências científicas devem ter muito mais prioridade na organização, sendo mantidas como centralidade nos processos decisórios.	"Tem que ter muito mais prioridade, para não baixar a priorização em relação ao que já é." (Participante 1)
Integração das evidências científicas na organização	Necessidade de integrar as evidências com maior frequência aos processos de trabalho, fortalecendo rotinas e promovendo aprendizado coletivo.	"As evidências científicas poderiam promover aperfeiçoamentos diversos; precisamos integrá-las aos processos de trabalho, que consequentemente demandam pesquisa e valorização. " (Participante 3)
Uso das evidências científicas	O uso das evidências é considerado inconsistente, variando entre áreas e dependendo de tempo e recursos disponíveis.	"Em alguns processos o uso está mais integrado, mas em outros não. Não há constância: algumas áreas usam com mais propriedade e outras não, seja por falta de tempo ou por não incorporar isso na rotina de trabalho. " (Participante 2)
Prioridades para aumentar a capacidade de pesquisa	Hierarquia de prioridades identificada: 1º melhor tempo de resposta; 2º equipe qualificada; 3º recursos financeiros; 4º incentivo; 5º especialistas externos.	"Não temos tempo hábil para produzir no ritmo que o gestor precisa. "(Participante 4) "Nosso problema não é só fazer acordos com especialistas externos, mas manter equipe qualificada, porque formamos as pessoas e a indústria contrata." (Participante 4) "Se tivéssemos recursos financeiros, poderíamos contratar instituições externas." (Participante 1) "O que menos precisamos agora é incentivo, porque vejo disposição interna para aumentar a capacidade de pesquisa." (Participante 3)
Obtenção de evidências científicas	Ordem de fontes mais utilizadas: 1º revistas especializadas; 2º bancos de dados; 3º literatura cinzenta; 4º aprendizado com colegas; 5º pesquisadores; 6º websites. Dificuldades de acesso e falta de padronização foram relatadas.	"Não há padronização de informação na organização; nem todos têm acesso às mesmas bases e bancos de dados. " (Participante 4) "O DataSUS não permite baixar artigos, eu baixo em casa. " (Participante 3)
Avaliação das evidências científicas	Necessidade de avaliar e adaptar as evidências com maior frequência, e não apenas utilizá-las de forma pontual.	"Precisamos avaliar e adaptar as evidências científicas com mais frequência. " (Participante 3)
Vinculação das evidências às principais questões enfrentadas	As evidências devem ser mais frequentemente consideradas nos processos de tomada de decisão em todos os níveis.	"É fundamental considerar as evidências científicas com maior frequência no processo de tomada de decisão. " (Participante 2)

## Discussão 

O presente estudo revelou que a SECTICS/MS apresenta uma capacidade institucional consolidada nas dimensões de adquirir e avaliar as evidências científicas para subsidiar a tomada de decisão. No entanto, foram identificadas fragilidades significativas nas dimensões "adaptar" e "aplicar", especialmente nos componentes de tradução do conhecimento e na comunicação entre áreas técnicas da instituição. A análise qualitativa apontou heterogeneidade entre departamentos no uso de evidências, com variações importantes na disponibilidade de equipe qualificada, tempo e acesso limitado a bases de dados especializadas. Além disso, observou-se baixa cooperação com especialistas externos, o que dificulta a aplicação prática do conhecimento científico. 

Considerando o momento do grupo focal, os participantes apontaram questões relevantes existente no Brasil como falta de tempo dos gestores, escassez de recursos para o financiamento de pesquisas e ausência do uso dos resultados das pesquisas para pautar as políticas, o que corrobora outros achados ^12^^,^^13^. De fato, os investimentos públicos em pesquisa e desenvolvimento foram reduzidos no país, e isso dificulta o desenvolvimento de novas pesquisas e a continuidade de muitas delas ^12^^-^^15^. Enquanto a Coreia do Sul e Israel, em 2023, investiram mais de 4% do seu produto interno bruto em pesquisa, a Europa aplicou cerca de 2%, os Estados Unidos 3% e o Brasil alocou apenas 1% ^16^. Cabe salientar, entretanto, que, em âmbito global, aproximadamente 80% dos países destinam menos de 1% do PIB a pesquisa e desenvolvimento (P&D), segundo o UNESCO Science Report 2021. Isso evidencia a expressiva desigualdade na distribuição mundial dos esforços de investimento científico ^17^. Tais elementos contribuem para uma percepção de uso inconsistente e pouco sistematizado das evidências científicas na formulação de políticas públicas.

A principal limitação deste estudo foi a baixa adesão à fase 2, com participação de apenas três dos cinco departamentos da SECTICS/MS, o que reduziu a abrangência das perspectivas. Além disso, a restrição do estudo exclusivamente à SECTICS/MS limitou a generalização dos resultados para outros órgãos do Ministério da Saúde e contextos institucionais. 

A seleção dos participantes foi feita por conveniência, por meio de indicação, o que pode ter gerado viés de seleção, restringindo a diversidade de perfis e opiniões. A forma de convite pode ter favorecido a participação de indivíduos mais próximos ou já engajados com a temática. Alternativas como realização de enquetes anônimo e inclusão de outros setores e instituições poderiam ter ampliado a adesão e a representatividade da amostra, o que possivelmente influenciaria os resultados obtidos. Também não houve análise longitudinal, o que limita a avaliação de mudanças ao longo do tempo. A natureza autoaplicável do instrumento pode ter gerado viés de resposta, como a desejabilidade social. Para mitigar esses efeitos, foram adotadas medidas como o anonimato das respostas, a clareza na formulação dos itens e a padronização das instruções para os participantes. Por fim, um participante não respondeu um item da dimensão "adquirir"; no entanto, essa resposta faltante não alterou o resultado desse item, pois os demais 11 participantes responderam "concordo" ou "concordo plenamente", atingindo um consenso de 92%.

Os achados são consistentes com estudos nacionais e internacionais que aplicaram a ferramenta 4A, os quais também indicam maior capacidade para adquirir evidências e dificuldades nas etapas "adaptar" e "aplicar" ^4^^,^^5^^,^^18^. Em 2013, instituições no Canadá apresentaram alta capacidade para adquirir evidências e moderada para avaliar e adaptar, porém enfrentaram desafios para aplicar as evidências na prática ^18^. Em Burkina Faso, Canadá e na Nigéria, nos anos de 2007, 2011 e 2015 respectivamente, essas capacidades foram limitadas em todas as dimensões, com desafios mais significativos em avaliação, adaptação e aplicação de evidências ^19^^-^^21^.

Em 2020, no Brasil, observou-se nos núcleos de evidências qualificação técnica adequada para adquirir e avaliar as evidências, mas também desafios para sua sustentabilidade, especialmente no fortalecimento da "adaptação" e da "aplicação" ^5^. 

A tradução do conhecimento é uma barreira persistente, especialmente em países de baixa e média renda, que enfrentam escassez de recursos humanos e financeiros ^19^, e a dificuldade de acesso às evidências (pesquisas e bases de dados fechadas), a escassez de equipe qualificada, pouca interação com pesquisadores e recursos insuficientes são os principais gargalos enfrentados nessa área ^22^. No entanto, há maior facilidade para implementar pesquisas quando se recebe treinamento sobre uso de evidências ^23^^,^^24^. 

Várias inciativas globais com reflexo no Brasil foram implementadas, a saber: 

Criou-se a rede EVIPNet em 2005, promovida pela Organização Mundial da Saúde, com o objetivo de facilitar o compartilhamento de boas práticas e a conexão entre pesquisadores e tomadores de decisão, tendo desdobramentos no Brasil em 2007, com a criação da rede para políticas informadas por evidências ^25^; 

Desenvolveram-se sínteses de evidências, pelas quais são produzidos resumos a partir de revisões sistemáticas, que traduzem o conhecimento científico para um formato acessível e aplicável a contextos locais, como a primeira síntese publicada em 2010 no Brasil ^26^; 

Fortaleceram-se capacidades institucionais, por meio de programas de capacitação e treinamento para profissionais de saúde e gestores em análise de evidências e aplicação prática, como o caso da especialização em políticas informadas por evidências no Brasil ^27^^,^^28^; 

Estabeleceram-se plataformas abertas de conhecimento, propiciando repositórios e bases de dados abertos para facilitar o acesso a informações científicas, como a Cochrane Library, a Health Evidence e a Health systems Evidence; 

Adotaram-se políticas governamentais, com foco no estabelecimento de normas e regulamentos que exigem o uso de evidências científicas na formulação e avaliação de políticas públicas, como a Lei n° 12.401 de 2011 para incorporação de tecnologias no SUS; 

Fomentaram-se pesquisas que conectem pesquisa científica diretamente com problemas de saúde pública. Essas iniciativas podem ser expandidas de modo a beneficiar todo o país ^23^^,^^24^.

A percepção nos achados quantitativos e qualitativos de que as evidências científicas, embora valiosas, ainda não são utilizadas de maneira suficientemente consistente ou frequente nos processos decisórios, reflete que essas limitações colocam a necessidade de maior investimento na institucionalização das políticas informadas por evidências e na tradução do conhecimento, de modo a promover estratégias para superar barreiras que dificultam a integração entre as evidências científicas, os gestores e a população.

A criação de um núcleo de apoio metodológico fortalece a sistematização e a comunicação das evidências científicas. O acesso ampliado a bases científicas e as ferramentas de síntese de evidências qualifica o uso do conhecimento. A integração intersetorial favorece o compartilhamento de boas práticas. Diretrizes formais para a tradução do conhecimento apoiam a adaptação das evidências ao contexto decisório. Essas estratégias podem contribuir para a institucionalização das políticas informadas por evidências. Investir na valorização da ciência e no fortalecimento de arranjos institucionais que promovam o uso de evidências em políticas públicas é fundamental para o fortalecimento do SUS. A continuidade de estudos nessa área contribuirá para consolidar práticas informadas em evidências, ampliando o impacto positivo na formulação e implementação de políticas públicas no Brasil.

## Data Availability

O banco de dados e os códigos de análise utilizados nesta pesquisa estão disponíveis mediante solicitação aos autores.
